# Probabilistic bias analysis for exposure misclassification of household income by neighbourhood in a cohort of individuals with colorectal cancer

**DOI:** 10.1093/ije/dyae135

**Published:** 2024-10-13

**Authors:** Laura E Davis, Hailey R Banack, Renzo Calderon-Anyosa, Erin C Strumpf, Alyson L Mahar

**Affiliations:** Department of Epidemiology, Biostatistics and Occupational Health, McGill University, Montreal, Quebec, Canada; Dalla Lana School of Public Health, University of Toronto, Toronto, Ontario, Canada; Department of Epidemiology, Biostatistics and Occupational Health, McGill University, Montreal, Quebec, Canada; Department of Epidemiology, Biostatistics and Occupational Health, McGill University, Montreal, Quebec, Canada; Department of Economics, McGill University, Montreal, Quebec, Canada; School of Nursing, Queens University, Kingston, Ontario, Canada; Department of Public Health Sciences, Queen’s University, Kingston, Ontario, Canada

**Keywords:** Area-level income, neighbourhood income, individual income, household income, quantitative bias analysis, exposure misclassification, income inequalities, cancer, colorectal cancer

## Abstract

**Introduction:**

Despite poor agreement, neighbourhood income is used as a proxy for household income, due to a lack of data availability. We quantified misclassification between household and neighbourhood income and demonstrate quantitative bias analysis (QBA) in scenarios where only neighbourhood income is available in assessing income inequalities on colorectal cancer mortality.

**Methods:**

This was a retrospective study of adults with colorectal cancer diagnosed 2006–14 from Statistics Canada’s Canadian Census Health and Environment Cohort. Neighbourhood income quintiles from Statistics Canada were used. Census household income quintiles were used to determine bias parameters and confirm results of the QBA. We calculated positive and negative predictive values using multinomial models, adjusting for age, sex and rural residence. Probabilistic QBA was conducted to explore the implication of exposure misclassification when estimating the effect of income on 5-year mortality.

**Results:**

We found poor agreement between neighbourhood and household income: positive predictive values ranged from 21% to 37%. The bias-adjusted risk of neighbourhood income on 5-year mortality was similar to the risk of mortality by household income. The bias-adjusted relative risk of the lowest income quintile compared with the highest was 1.42 [95% simulation interval (SI) 1.32–1.53] compared with 1.46 [95% confidence interval (CI) 1.39–1.54] for household income and 1.18 (95% CI 1.12–1.24) for neighbourhood income.

**Conclusion:**

QBA can be used to estimate adjusted effects of neighbourhood income on mortality which represent household income. The predictive values from our study can be applied to similar cohorts with only neighbourhood income to estimate the effects of household income on cancer mortality.

Key Messages

- This study investigates the magnitude of misclassification that arises when neighbourhood income quintile is used instead of household income quintile and demonstrates the application of quantitative bias analysis to present adjusted estimates for the risk of 5-year mortality in colorectal cancer.
- We found very poor agreement between neighbourhood and household income, and adjustment of neighbourhood income using quantitative bias analysis resulted in similar relative risks to household income.
- The methods presented here can be replicated in different scenarios, and bias parameters from our study can be applied to similar cohorts to estimate the effects of household income on cancer mortality when only neighbourhood income is available.

## Introduction

Income, whether measured at the individual, household or area level, is a key social determinant of health, affecting outcomes ranging from health behaviours to diabetes to cancer.^1^^,^^2^ Individual income, often measured as household income, will affect the individual’s ability to access material goods and services. Area-level income refers to the average or median income in a census tract, dissemination area or other geographical region, and effects health through features of the physical environment, such as easier access to primary care or neighbourhood social support.^3^^,^^4^ Due to limitations on data availability, health services researchers and social epidemiologists using routinely collected data often use neighbourhood income as a proxy for household-level income.^5–7^ Studies investigating cancer, health behaviours, chronic disease and child health across Canada, the USA and the UK have all demonstrated that neighbourhood income is a poor proxy for household income, with poor agreement and less variation in neighbourhood than household income.^6^^,^^8–12^ Consequently, using neighbourhood income as a substitute for household income may underestimate the impact of income on health outcomes and can lead to incorrect conclusions regarding income inequalities.^9^^,^^10^^,^^13–16^

Quantitative bias analysis (QBA) is a simple but underused method to obtain estimates of the direction, magnitude and uncertainty arising from exposure misclassification.^17–19^ To date, this method has rarely been used in scenarios with polytomous exposures to quantify misclassification at each level of a categorical variable.^20^ We describe an approach to address the common practice of using neighbourhood income quintile in the absence of household income quintile in a five-by-five scenario when evaluating income as an exposure. In a cohort of individuals with colorectal cancer (CRC) we aimed to: (i) investigate the potential magnitude of exposure misclassification when neighbourhood is used instead of household income quintile; and (ii) demonstrate the application of QBA in scenarios where only neighbourhood income quintile is available in assessing inequalities in the risk of mortality.

## Methods

### Study design and population

**Figure 1.**
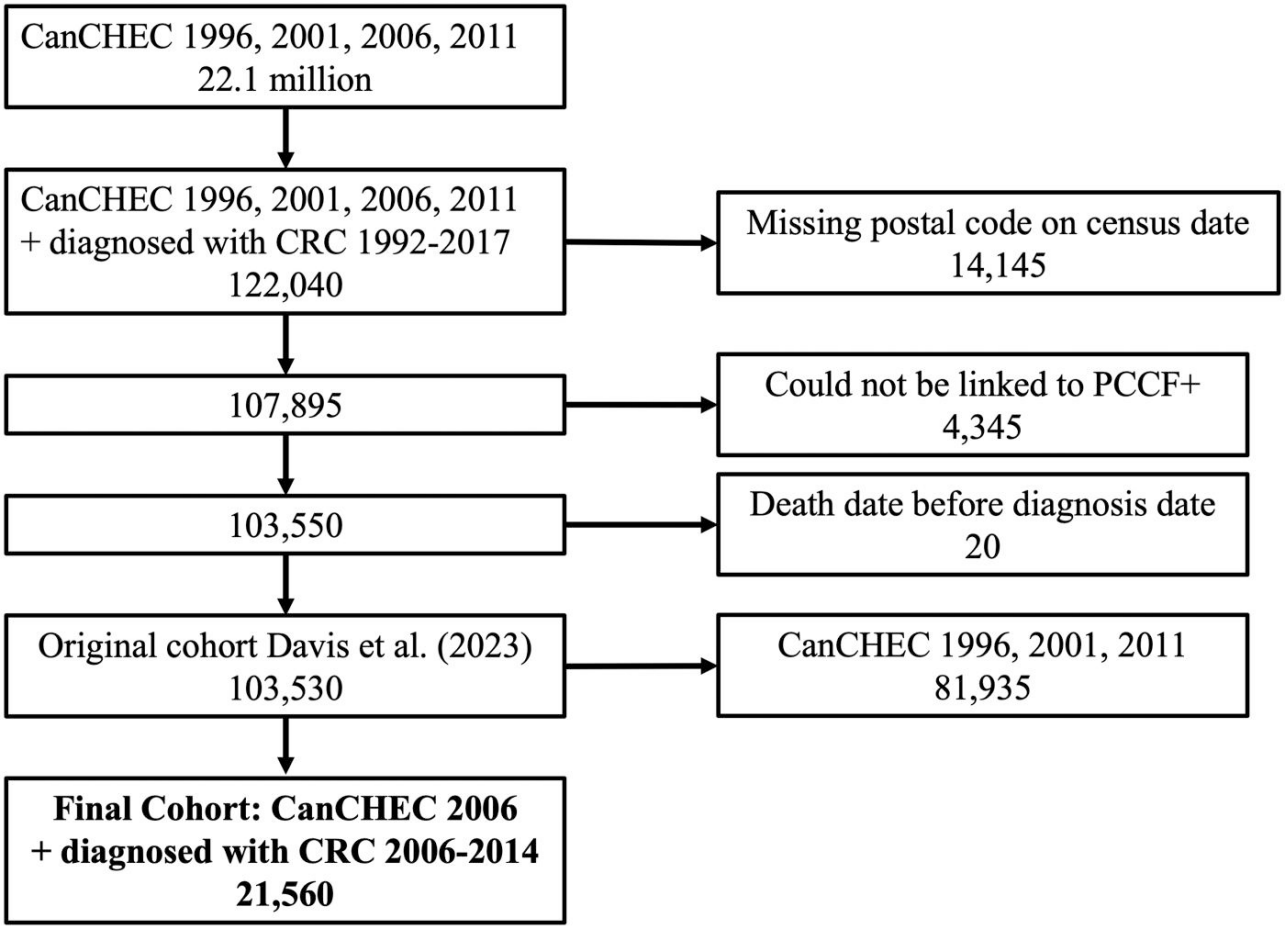
Cohort creation. CanCHEC, Canadian Census Health and Environment Cohorts; CRC, Colorectal cancer; PCCF+, Postal code conversation file plus

This was a retrospective cohort study using the Canadian Census Health and Environment Cohorts (CanCHEC).^10^^,^^21^ CanCHEC is a nationwide cohort created by Statistics Canada, which integrates census data, vital statistics and cancer registry databases.^21^ The census is performed every 5 years in Canada, and approximately one in every five households completes the long-form census. The Canadian Cancer Registry (CCR) collects cancer data on all Canadians diagnosed with cancer, except Quebec residents after 2010.^22^ The postal code conversation file (PCCF+) was linked to the CanCHEC using the participants’ postal code at census to obtain neighbourhood income quintiles and rural residence. We used a subset of individuals with CRC from the CanCHEC created for a previous study which is described in more detail elsewhere.^10^ Briefly, the previous study included individuals >35 with a new diagnosis of CRC between 1992 and 2017. For this study, we further limited the cohort to individuals who responded to the 2006 census and had a CRC diagnosis between 1 January 2006, and 31 December 2014 (Figure 1). The 2006 census was the first census to use tax files to measure household income. We additionally excluded 40 individuals residing in the Territories due to small sample sizes.

Ethics approval was obtained from the McGill University Research Ethics Board (#A04-M37-22A) and Statistics Canada.

### Exposure

We had household and neighbourhood income quintile measures for all individuals in the cohort. Household income was defined as the true exposure and neighbourhood income as misclassified household income. Both household and neighbourhood income were measured at the time and residence of the 2006 census and represented income before the cancer diagnosis.

*Neighbourhood income quintiles* were created by Statistics Canada for the PCCF+ and are the most widely used area-level income measure in Canada.^23^ Total income for each dissemination area (DA), Canada’s smallest geographical area, is calculated by multiplying the median before-tax income of that area by the total number of households and adjusted for household size by dividing by the sum of the single-person equivalents of the DA to obtain the median household income per single-person equivalent for each DA.^24^^,^^25^ Census metropolitan area (CMA) and census agglomeration (CA) quintiles are then constructed by ranking DAs within each CMA/CA/other region by province from lowest to highest, then dividing into fifths.^26^^,^^27^ CMA/CA specific quintiles take into account differences in cost of living across regions. We linked the PCCF+ to the CanCHEC using the postal code in 2006.

*Household income quintile* was created to be as similar as possible to neighborhood income and defined as adjusted before-tax household income using the long-form census. All sources of income from the calendar year before the census (2005) were summed for each household and adjusted for the number of household members, using the single-person equivalence scales from Statistics Canada’s low-income cut-offs.^28^ In 2006, individuals responding to the census had the option to consent to income tax linkage. All individuals included in the cohort consented to the use of income tax linkage. CMA/CA/other region household income quintiles were created in the same way as neighbourhood income quintiles.

### Outcome

Five-year mortality was dichotomized as death from any cause from the vital statistics database occurring within 5 years from diagnosis. The study period allowed for 5 years of complete follow-up on all individuals.

### Covariates

Predictors of misclassification of household income by neighbourhood income quintiles were conceptualized as rural residence, age and sex. Previous work has demonstrated that larger rural areas suffer from greater misclassification compared with smaller urban areas.^10^ Rural residence was defined as residing in a census subdivision with a population of <1000 and a population density of <400 persons per square kilometre. Continuous age and dichotomous sex were defined according to the census. We stratified results by province/territory at census to obtain bias parameters that could be applicable by province/territory. Due to small sample sizes, we grouped Prairie provinces (Alberta, Manitoba and Saskatchewan), and Atlantic provinces (New Brunswick, Nova Scotia, Prince Edward Island and Newfoundland). Additionally, we described tumour location (colon or rectum) and stage at diagnosis, from the CCR.^29^^,^^30^

### Statistical analysis

**Figure 2.**
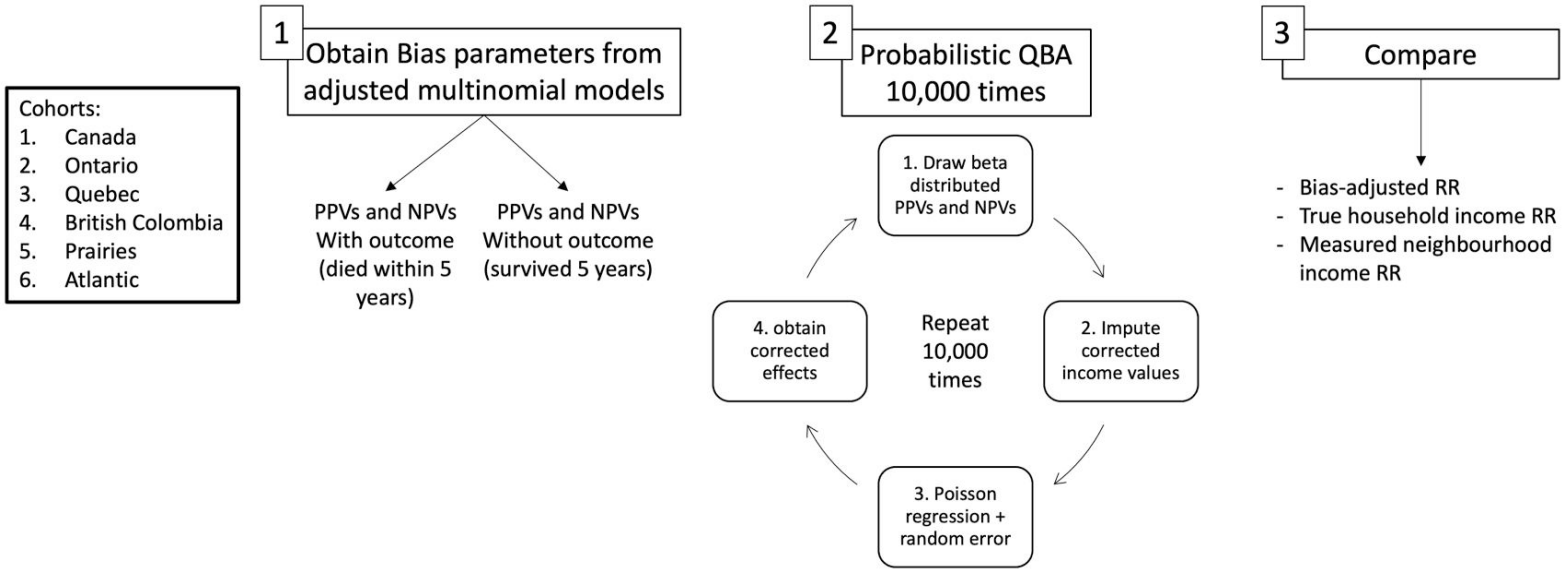
Analysis steps. PPVs, Positive predictive values; NPVs, Negative predictive values; QBA, quantitative bias analysis; RR, relative risk

The analysis involved three main steps. First, we calculated the bias parameters (positive and negative predictive values) separately for those who survived and did not survive 5 years, to determine the probability of being truly classified in household income quintiles 1–5 given the observed classification of neighbourhood income quintiles 1–5.^31^ Second, we performed probabilistic QBA to obtain bias-adjusted measures of the effect of neighbourhood income on 5-year mortality. Third, we compared the bias-adjusted estimates with the effect of household income on 5-year mortality (considered correct) and the misclassified neighbourhood income estimates (considered incorrect). We performed each of these steps within the Canadian CRC cohort and stratified by province of residence. These steps are defined in more detail in Figure 2 and below.

R Studio version 4.2.2 was used for all analyses and detailed code is provided at [https://github.com/ldavis020/QBA-income]. In keeping with Statistics Canada’s data confidentiality guidelines, counts were rounded to the nearest five.

### Step 1: obtain bias parameters

We extended the traditional dichotomous scenario to calculate positive and negative predictive values for each combination of quintiles (25 values total), stratified by 5-year mortality.^19^ Positive predictive values (PPVs) represent the probability of being correctly classified in household income quintiles 1–5 given the same classification by neighbourhood income quintiles (five values). Negative predictive values (NPVs) indicate the probability of being truly classified in household income quintiles 1–5, despite an incorrect classification of observed neighbourhood income quintile for each of the remaining four quintiles (20 values). Supplementary Table S1 (available as Supplementary data at *IJE* online) provides an example of how to calculate crude predictive values.

Extending previous methods for dichotomous scenarios, we calculated negative and positive predictive values from multinomial logistic regression models, stratified by 5-year mortality.^32^^,^^33^ Multinomial models allow for the adjustment of predictors of misclassification which were age, sex and rural residence.^32^^,^^33^ To calculate PPVs and NPVs using this model, we defined the true exposure (household income quintile) as the outcome and the misclassified exposure (neighbourhood income quintile) as the predictor for those who died within 5 years and those who survived.^19^^,^^32^ Using the model coefficients, we predicted the probability that household income quintile corresponded to neighbourhood income quintile while holding all covariates at their mean.^34^ Adjusted PPVs and NPVs were calculated for the whole Canadian cohort and by province (Quebec, Ontario, British Colombia, Prairie provinces and Atlantic provinces).

### Step 2: probabilistic quantitative bias analysis

We used the PPVs and NPVs from Step one to conduct a probabilistic record-level QBA with Monte Carlo sampling to adjust for exposure misclassification.^19^^,^^20^^,^^33^ We performed the QBA in the whole Canadian cohort and stratified by province.

In Step 2.1 we used a beta distribution to incorporate uncertainty in the predictive values obtained in Step 1.^19^^,^^33^ The beta distribution can model a wide range of probability density shapes, which is ideal for proportions and does not yield values outside of an allowed range, such as with the normal distribution.^19^ We parameterized the beta distribution by choosing upper and lower values of the predictive values that are likely with only 2.5% probability of seeing a value lower than the low end and 2.5% probability of seeing a value higher than the high end of the range.^19^ The ends of the range (U and L) and the predictive values (x) are used in the following equations to obtain alpha and beta values.^19^ We defined U and L as the lower and upper values of the 95% confidence intervals from the adjusted PPVs and NPVs.


$$\begin{array}{c} sd=\frac{U-L}{2\cdot 1.96}\\ \alpha =x\;\left(\frac{x(1-x)}{sd^{2}}-1\right)\\ \beta =(1-x)\left(\frac{x(1-x)}{sd^{2}}-1\right) \end{array}$$

In Step 2.2, we imputed the adjusted income quintiles for each combination of neighbourhood income quintiles and the outcome for each observation in the dataset (10 possible combinations). We used the probability distribution of the predictive values obtained from Step 2.1 to reassign each neighbourhood income quintile into the bias-adjusted income quintile. For example, an individual observed to be in neighbourhood income quintile 1 would be reassigned based on the sampled probabilities (PPVs and NPVs) for household income quintile 1–5. This step creates a new dataset with the imputed bias-adjusted values for income.

In Step 2.3, we used the bias-adjusted dataset to estimate the association between income and 5-year mortality using Poisson regression with robust error variance to obtain relative risks.^35^^,^^36^ We present unadjusted and adjusted models for age, sex and rural residence. We accounted for total study error by incorporating random error into each of the bias-adjusted effect estimates by adding a number drawn randomly from a standard normal distribution.^19^ Steps 2.1 to 2.3 were repeated 10 000 times to create a distribution of relative risks. The bias-adjusted effect estimates are the 50th percentile of the distribution and the 2.5th and 97.5th percentile of the distribution provides a 95% simulation interval (SI).

### Step 3: Effect of household and neighbourhood income on 5-year mortality

We compared the bias-adjusted effects of neighbourhood income with the true effect of household income on 5-year mortality, using unadjusted and adjusted Poisson regression with robust error variance to obtain relative risks and 95% confidence intervals (CI). Adjusted models included age, sex and rural residence.

## Results

**Figure 3.**
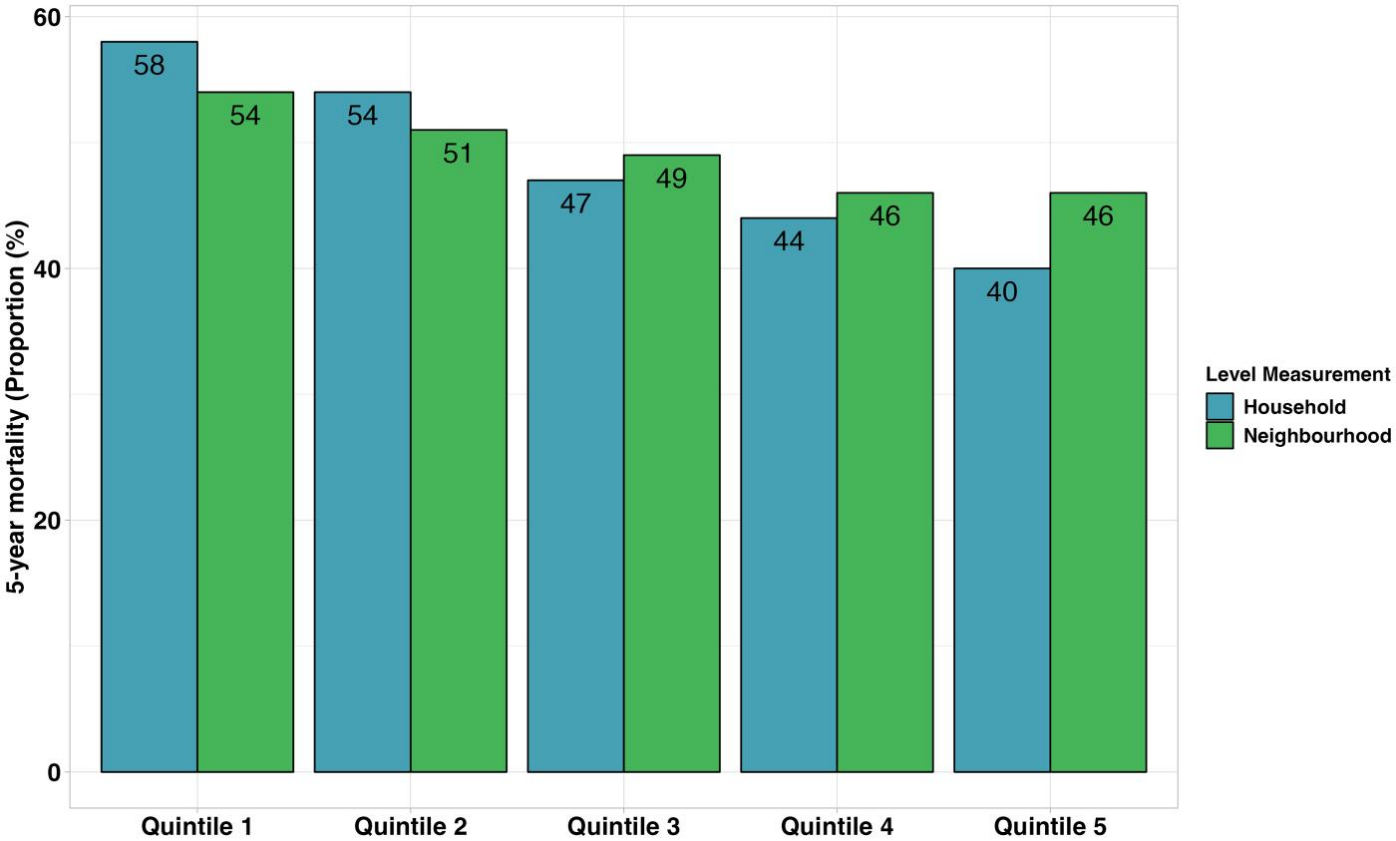
Proportion of those who died within 5 years by household and neighbourhood income quintiles. Quintile 1 = lowest income; Quintile 5 = highest income

The cohort included 21 560 individuals with CRC diagnosed between 2006 and 2014. Cohort characteristics are described in Table 1 for the overall cohort and by household and neighbourhood income quintile. Individuals without postal codes were more likely to have low household income but were excluded because neighbourhood income could not be determined (Supplementary Table S2, available as Supplementary data at *IJE* online). The mean age was 66 years, 55.3% were male, 23.7% lived in rural areas and most individuals resided in Ontario (40.6%) followed by Quebec (15.9%); 49.2% of individuals died within 5 years. The proportion of those who died within 5 years by household and neighbourhood income quintile is described in Figure 3. Five-year mortality was 58% and 54% for individuals in the lowest neighbourhood and household income quintile, compared with 40% and 46% in the highest neighbourhood and household income quintile.

**Table 1.** Cohort characteristics by household and neighbourhood income quintiles^a^

		Household income quintile					Neighbourhood income quintile				
Variable	Overall (*n *=* *21 5600)	Q1 (*n *=* *4235)	Q2 (*n *=* *4925)	Q3 (*n *=* *4300)	Q4 (*n *=* *4065)	Q5 (*n *=* *4040)	Q1 (*n *=* *4210)	Q2 (*n *=* *4485)	Q3 (*n *=* *4385)	Q4 (*n *=* *4245)	Q5 (*n *=* *4235)
Age at diagnosis, years, mean (SD)	65.96 (12.46)	68.72 (12.85)	68.67 (12.06)	65.69 (12.32)	63.80 (11.98)	62.25 (11.73)	66.91 (12.68)	66.67 (12.28)	65.88 (12.40)	64.90 (12.54)	65.42 (12.32)
Sex											
Male	11 930 (55.3)	1845 (43.5)	2715 (55.1)	2480 (57.7)	2395 (58.9)	2495 (61.8)	2225 (52.9)	2435 (54.3)	2410 (55.0)	2405 (56.7)	2450 (57.8)
Female	9635 (44.7)	2390 (56.5)	2215 (44.9)	1820 (42.3)	1670 (41.1)	1545 (38.2)	1985 (47.1)	2050 (45.7)	1970 (45.0)	1840 (43.3)	1790 (42.2)
Tumour location											
Rectal	7230 (33.5)	1410 (33.3)	1570 (31.9)	1465 (34.1)	1390 (34.3)	1390 (34.5)	1425 (33.8)	1485 (33.1)	1500 (34.3)	1420 (33.4)	1405 (33.2)
Colon	14 330 (66.5)	2820 (66.7)	3355 (68.1)	2830 (65.9)	2670 (65.7)	2645 (65.5)	2790 (66.2)	3000 (66.9)	2880 (65.7)	2825 (66.6)	2830 (66.8)
Rural residence											
Not rural	16 440 (76.3)	3180 (75.1)	3660 (74.3)	3345 (77.8)	3170 (78.0)	3090 (76.5)	3315 (78.8)	3445 (76.8)	3310 (75.6)	3165 (74.5)	3205 (75.7)
Rural	5120 (23.7)	1055 (24.9)	1270 (25.7)	955 (22.2)	895 (22.0)	950 (23.5)	895 (21.2)	1040 (23.2)	1070 (24.4)	1080 (25.5)	1030 (24.3)
Province at diagnosis											
Newfoundland and Labrador	650 (3.0)	135 (3.2)	145 (3.0)	135 (3.2)	110 (2.8)	120 (3.0)	140 (3.3)	140 (3.1)	130 (2.9)	115 (2.7)	130 (3.0)
PEI	125 (0.6)	25 (0.6)	20 (0.4)	20 (0.4)	30 (0.7)	30 (0.7)	20 (0.5)	15 (0.4)	30 (0.7)	30 (0.8)	30 (0.6)
Nova Scotia	945 (4.4)	180 (4.3)	215 (4.4)	190 (4.4)	185 (4.6)	175 (4.3)	185 (4.4)	195 (4.4)	205 (4.7)	185 (4.3)	175 (4.1)
New Brunswick	630 (2.9)	150 (3.5)	145 (2.9)	125 (3.0)	105 (2.6)	105 (2.6)	150 (3.5)	130 (2.9)	130 (2.9)	105 (2.5)	115 (2.7)
Quebec	3420 (15.9)	755 (17.8)	820 (16.6)	625 (14.6)	600 (14.8)	620 (15.4)	725 (17.2)	730 (16.3)	725 (16.6)	615 (14.5)	620 (14.7)
Ontario	8750 (40.6)	1600 (37.8)	2035 (41.3)	1760 (40.9)	1690 (41.6)	1670 (41.3)	1615 (38.4)	1815 (40.4)	1750 (39.9)	1825 (43.0)	1745 (41.2)
Manitoba	1020 (4.7)	180 (4.3)	220 (4.5)	220 (5.1)	195 (4.8)	210 (5.1)	170 (4.1)	210 (4.6)	240 (5.4)	205 (4.8)	200 (4.7)
Saskatchewan	910 (4.2)	170 (4.1)	215 (4.3)	200 (4.7)	160 (4.0)	160 (4.0)	195 (4.7)	185 (4.2)	185 (4.2)	160 (3.8)	180 (4.3)
Alberta	2055 (9.5)	490 (11.6)	420 (8.5)	430 (10.0)	365 (9.0)	345 (8.6)	405 (9.6)	435 (9.7)	405 (9.2)	375 (8.8)	435 (10.2)
British Columbia	3055 (14.2)	550 (13.0)	695 (14.1)	590 (13.8)	620 (15.3)	600 (14.9)	605 (14.4)	625 (13.9)	590 (13.5)	625 (14.7)	610 (14.4)
Diagnosis year											
2006	2620 (12.2)	530 (12.6)	665 (13.5)	490 (11.4)	475 (11.7)	455 (11.3)	515 (12.3)	560 (12.5)	550 (12.5)	485 (11.4)	510 (12.1)
2007	2740 (12.7)	605 (14.3)	660 (13.4)	495 (11.5)	475 (11.7)	510 (12.6)	540 (12.8)	575 (12.8)	545 (12.5)	565 (13.3)	515 (12.2)
2008	2955 (13.7)	615 (14.6)	695 (14.1)	600 (14.0)	525 (13.0)	515 (12.7)	605 (14.4)	630 (14.1)	605 (13.8)	565 (13.4)	550 (12.9)
2009	2890 (13.4)	570 (13.4)	605 (12.3)	585 (13.7)	570 (14.0)	560 (13.9)	560 (13.3)	595 (13.3)	575 (13.1)	575 (13.6)	580 (13.7)
2010	3000 (13.9)	595 (14.0)	685 (13.9)	595 (13.8)	575 (14.2)	545 (13.5)	620 (14.7)	605 (13.5)	595 (13.6)	575 (13.6)	600 (14.2)
2011	1795 (8.3)	320 (7.5)	430 (8.7)	370 (8.6)	335 (8.2)	345 (8.5)	330 (7.8)	355 (7.9)	370 (8.4)	375 (8.9)	360 (8.5)
2012	1865 (8.7)	350 (8.3)	400 (8.1)	395 (9.1)	350 (8.6)	370 (9.2)	340 (8.1)	385 (8.6)	390 (8.9)	385 (9.1)	360 (8.5)
2013	1890 (8.8)	335 (8.0)	410 (8.4)	385 (9.0)	385 (9.4)	375 (9.2)	330 (7.8)	415 (9.3)	400 (9.1)	370 (8.7)	375 (8.8)
2014	1805 (8.4)	315 (7.4)	375 (7.6)	380 (8.9)	375 (9.2)	365 (9.1)	370 (8.8)	360 (8.0)	355 (8.1)	340 (8.0)	380 (9.0)
Stage at diagnosis											
0	80 (0.4)	10 (0.2)	15 (0.3)	20 (0.5)	20 (0.5)	15 (0.4)	15 (0.4)	15 (0.3)	20 (0.4)	15 (0.3)	15 (0.3)
I	2030 (9.4)	340 (8.0)	420 (8.5)	445 (10.3)	400 (9.8)	430 (10.6)	365 (8.7)	425 (9.5)	405 (9.0)	420 (9.4)	415 (9.3)
II	2395 (11.1)	450 (10.6)	560 (11.4)	505 (11.7)	455 (11.2)	425 (10.5)	465 (11.0)	510 (11.4)	470 (10.5)	480 (10.7)	475 (10.6)
III	2570 (11.9)	445 (10.5)	530 (10.8)	525 (12.2)	535 (13.2)	535 (13.2)	480 (11.4)	530 (11.8)	540 (12.0)	515 (11.5)	505 (11.3)
IV	1790 (8.3)	355 (8.4)	415 (8.4)	355 (8.3)	340 (8.4)	325 (8.0)	350 (8.3)	355 (7.9)	370 (8.3)	345 (7.7)	375 (8.4)
Unknown	455 (2.1)	105 (2.5)	120 (2.4)	85 (2.0)	75 (1.8)	75 (1.9)	105 (2.5)	85 (1.9)	90 (2.0)	90 (2.0)	90 (2.0)
Missing	12 240 (56.8)	2530 (59.7)	2864 (58.2)	2365 (55.0)	2240 (55.1)	2560 (63.4)	2430 (57.7)	2565 (57.2)	2490 (55.5)	2380 (53.1)	2360 (52.6)
Household income quintile											
Quintile 1	4235 (19.6)	4235 (100.0)	0 (0.0)	0 (0.0)	0 (0.0)	0 (0.0)	1440 (34.2)	955 (21.3)	790 (18.1)	600 (14.1)	445 (10.6)
Quintile 2	4930 (22.9)	0 (0.0)	4925 (100.0)	0 (0.0)	0 (0.0)	0 (0.0)	1130 (26.8)	1190 (26.5)	1060 (24.2)	845 (20.0)	700 (16.5)
Quintile 3	4300 (19.9)	0 (0.0)	0 (0.0)	4300 (100.0)	0 (0.0)	0 (0.0)	735 (17.4)	945 (21.1)	930 (21.2)	920 (21.6)	770 (18.2)
Quintile 4	4065 (18.8)	0 (0.0)	0 (0.0)	0 (0.0)	4065 (100.0)	0 (0.0)	555 (13.1)	805 (17.9)	870 (19.8)	940 (22.2)	895 (21.2)
Quintile 5	4040 (18.7)	0 (0.0)	0 (0.0)	0 (0.0)	0 (0.0)	4040 (100.0)	360 (8.5)	590 (13.2)	730 (16.7)	940 (22.1)	1420 (33.5)
Neighbourhood income quintile											
Quintile 1	4210 (19.5)	1440 (34.0)	1130 (22.9)	735 (17.1)	555 (13.6)	360 (8.9)	4210 (100.0)	0 (0.0)	0 (0.0)	0 (0.0)	0 (0.0)
Quintile 2	4485 (20.8)	955 (22.6)	1190 (24.1)	945 (22.0)	805 (19.8)	590 (14.6)	0 (0.0)	4485 (100.0)	0 (0.0)	0 (0.0)	0 (0.0)
Quintile 3	4385 (20.3)	790 (18.7)	1060 (21.5)	930 (21.7)	870 (21.4)	730 (18.1)	0 (0.0)	0 (0.0)	4385 (100.0)	0 (0.0)	0 (0.0)
Quintile 4	4245 (19.7)	600 (14.2)	845 (17.2)	920 (21.4)	940 (23.2)	940 (23.2)	0 (0.0)	0 (0.0)	0 (0.0)	4245 (100.0)	0 (0.0)
Quintile 5	4235 (19.6)	445 (10.6)	700 (14.2)	770 (17.9)	895 (22.1)	1420 (35.2)	0 (0.0)	0 (0.0)	0 (0.0)	0 (0.0)	4235 (100.0)

CI, confidence interval; PEI, Prince Edward Island; Q, quintile; Q1, lowest income; Q5, highest income.

a Figures are *n* (column %).

The PPVs and NPVs for Canada are presented in Table 2 and by province in Supplementary Table S3 (available as Supplementary data at *IJE* online). Crude and adjusted bias parameters were similar. Overall, PPVs were very low, ranging from 20.64% (95% CI 18.75–22.68) to 36.93% (95% CI 35.05–38.84). Compared with those who died within 5 years, individuals who survived 5 years had higher PPVs at the highest income quintile (36.93% vs 27.69%) and lower values at the lowest income quintile (31.27% vs 36.62%).

**Table 2.** Adjusted predictive values and 95% CIs stratified by 5-year mortality for all of Canada^a^

	Household income quintile									
Neighbourhood income quintile	Count					Adjusted predictive value (95% CI)				
	Q1	Q2	Q3	Q4	Q5	Q1	Q2	Q3	Q4	Q5
**Survived 5 years (*n *=* *12 445)**										
Q1	710	590	405	325	205	31.27 (29.36–33.26)	26.51 (24.70–28.41)	18.57 (17.00–20.26)	14.65 (13.23–16.19)	8.99 (7.87–10.25)
Q2	455	615	560	490	395	17.62 (16.17–19.17)	24.14 (22.49–25.87)	22.78 (21.17–24.49)	19.84 (18.30–21.46)	15.62 (14.24–17.11)
Q3	415	570	560	540	470	15.78 (14.41–17.25)	22.28 (20.70–23.96)	22.49 (20.89–24.16)	21.32 (19.76–22.97)	18.13 (16.68–19.69)
Q4	310	465	570	595	635	11.90 (10.70–13.22)	17.99 (16.54–19.54)	22.76 (21.16–24.43)	23.36 (21.75–25.05)	24.00 (22.37–25.70)
Q5	220	370	455	560	950	8.36 (7.35–9.50)	14.27 (12.96–15.69)	18.19 (16.73–19.75)	22.25 (20.66–23.92)	36.93 (35.05–38.84)
**Died within 5 years (*n *=* *9150)**										
Q1	730	540	330	230	150	36.62 (34.48–38.80)	27.56 (25.61–29.60)	16.83 (15.23–18.57)	11.52 (10.17–13.01)	7.47 (6.40–8.72)
Q2	500	575	385	315	195	24.80 (22.93–26.78)	29.45 (27.46–31.52)	20.01 (18.28–21.85)	16.04 (14.47–17.74)	9.70 (8.47–11.09)
Q3	380	490	370	330	260	20.30 (18.51–22.23)	26.91 (24.90–29.01)	20.76 (18.94–22.70)	18.16 (16.44–20.01)	13.88 (12.36–15.54)
Q4	285	380	345	345	305	17.08 (15.33–18.98)	23.34 (21.34–25.46)	21.37 (19.44–23.43)	20.64 (18.75–22.68)	17.57 (15.84–19.48)
Q5	230	330	315	335	470	13.12 (11.58–14.82)	19.65 (17.80–21.63)	19.25 (17.41–21.22)	20.30 (18.43–22.31)	27.69 (25.57–29.91)

CI, confidence interval; Q, quintile; Q1, lowest income; Q5, highest income.

a Predictive values and CIs obtained from multinomial models. Predictive values adjusted for age, sex and rural residence.

Household income had a greater estimated effect on 5-year mortality compared with neighbourhood income, both in Canada overall and across provinces. For example, individuals experiencing the lowest household income had a 46% (95% CI 1.39–1.54) increase in the risk of 5-year mortality compared with those experiencing the highest income, whereas those living in neighbourhoods with the lowest income had only an 18% (95% CI 1.12–1.24) increased risk of 5-year mortality compared with those living in neighbourhoods with the highest income.

**Figure 4.**
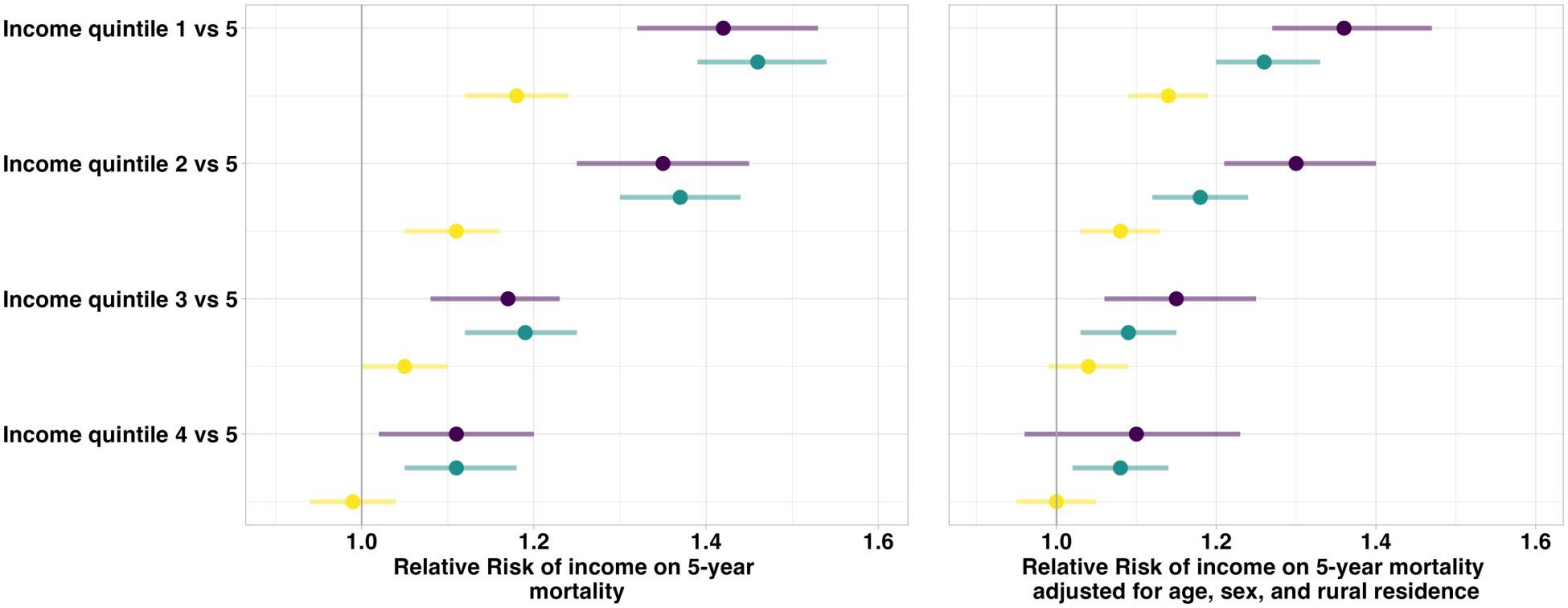
Unadjusted (left) and adjusted (right) bias-adjusted relative risks of death within five years (yes/no) with systematic and random error and 95% simulation intervals for all of Canada, compared with the relative risk of death for true household income and measured neighbourhood income. The reference category is the highest income quintile for all comparisons (quintile 5). QBA, Quantitative bias analysis

The results of the bias analysis are presented in Figure 4 and Supplementary Table S4 (available as Supplementary data at *IJE* online). After accounting for both random and systematic error, the bias-adjusted 5-year mortality risk was similar to the risk of mortality by household income, with overlapping confidence intervals. For example, the bias-adjusted relative risk of being in the lowest income quintile compared with the highest for Canada was 1.42 (95% SI 1.32–1.53) compared with 1.46 (95% CI 1.39–1.54) for household income and 1.18 (95% CI 1.12–1.24) for neighbourhood income. After adjusting for age, sex and rural residence, bias-adjusted effects of income were greater than the adjusted effect of household income on 5-year mortality. This pattern persisted across provinces, with all provinces demonstrating similar bias-adjusted effects compared with household income.

## Discussion

Using routinely collected administrative data from a representative sample of Canadians with CRC, we provide detailed methodology on how to use QBA to estimate bias-adjusted effects of neighbourhood income on 5-year mortality. By comparing bias-adjusted estimates with the effect of household income and neighbourhood income on 5-year mortality, we were able to demonstrate that the QBA provided similar effects to household income. This is one of few studies detailing how to obtain bias parameters from multinomial models for multicategorical variables and apply them to QBA.^20^

Our study is in line with previous studies that have found poor agreement between individual or household and neighbourhood income quintiles, with agreement ranging from 34% to 37%.^9^^,^^10^^,^^37^ Other solutions to the problem of limited access to individual- or household-level income variables have been proposed. For example, researchers have developed a housing-based socioeconomic index (HOUSES) that correlated strongly with other socioeconomic factors, such as household income and education levels.^38^^,^^39^ This method has been used to describe socioeconomic inequalities in asthma research, vaccine research and child and youth health outcomes.^39–41^ Another study in the USA developed a method of approximating household-level income using income probabilities from the US census.^42^ However, both these methods require access to census data or other individual-level housing information, which is not always available. Our method allows researchers to use the PPVs and NPVs presented in our study to obtain bias-adjusted effects of income on 5-year mortality in cohorts with similar prevalence of exposure, covariates and predictors of misclassification.

### Implications and future directions

With increasing calls to understand socioeconomic inequities in the health care system, and in cancer care specifically, it is imperative that studies examine both household and neighbourhood-level income inequalities.^43–45^ Although the lack of individual income data is a challenge faced by many researchers who use administrative health data, this analysis was conducted using data from Canada, which may affect the generalizability of our findings. However, the methods presented in this analysis are broadly applicable. The PPVs and NPVs could be applied directly to perform QBA in CRC cohorts outside of Canada with similar prevalence of exposure, outcomes, covariates and predictors of misclassification, to determine the effect of household income on 5-year mortality, when only neighbourhood income is available. Second, the detailed methodology and code provided can be used to replicate our bias analysis in different contexts when personal or household income is the main exposure but individual measures are not available. For example, future research might aim to broaden the scope of this study by examining misclassification of income in other jurisdictions outside of Canada or in other disease sites.

### Strengths

We used a measurement of neighbourhood income that is broadly used across Canada and we present a method for understanding the effects of household income on colorectal cancer mortality when only neighbourhood income is available. By having both household and neighbourhood income for all individuals, we were able to demonstrate the validity of using QBA to provide an adjusted estimate of the effect of income on mortality. Moreover probabilistic QBA, including the use of a multivariable model to estimate predictive values, is preferable to other methods of misclassification adjustment, such as missing data methods which are constrained to the situations where internal validation data are the source of bias parameters.^31^ This is also one of the few studies that demonstrate the use of QBA for a multicategorical exposure.

### Limitations

Our study has some limitations. First, we used negative and positive predictive values, which need to be calculated separately for each outcome; therefore whereas our bias parameters can be applied to cohorts with similar prevalence of exposure, covariates and predictors of misclassification, they can only be used for studies examining income and 5-year mortality. Moreover, we did not have access to continuous neighbourhood income for the entire study period. Future research might examine misclassification of income on the continuous scale, where regression calibration might be applied. For simplicity, we examined 5-year mortality instead of survival. Since many cancer studies examine survival, this may limit the generalizability of our work. Next, the predictive values from our validation study can only be applied to external cohorts that have a similar prevalence of exposure, covariates and predictors of misclassification; therefore, our results are not generalizable outside of Canada or to other cancer sites. More work needs to be done to calculate predictive values in other cancer sites or to determine if predictive values for all Canadians can be applied to subgroups such as individuals with cancer. Finally, we excluded a significant number of individuals with missing postal codes, resulting in a cohort with fewer individuals experiencing low income. However, we compared our cohort with individuals diagnosed within the same time frame in the CCR, and the cohorts were similar (Supplementary Table S5, available as Supplementary data at *IJE* online).

## Conclusion

Ideally all routinely collected administrative databases used for research would include both area-level and individual-level socioeconomic variables, such as individual or household income quintiles from tax records. Until then, our study provides a relatively simple method to estimate the effect of household income on cancer mortality when only neighbourhood income is available. Importantly, we provide foundational methodological processes for future studies to replicate our work in other diseases sites and countries where income is the primary exposure of interest.

## Ethics approval

Ethics approval was obtained from the McGill University Research Ethics Board (#A04-M37-22A) and Statistics Canada.

## Supplementary Material

dyae135_Supplementary_Data

## Data Availability

The CanCHEC database is protected by Statistics Canada confidentiality policies and cannot be made publicly available. Access may be granted to those who meet prespecified criteria for confidential access, available at [https://www.statcan.gc.ca/en/microdata/data-centres/access].

## References

[1] Kawachi I , Kennedy BP. Socioeconomic determinants of health : health and social cohesion: why care about income inequality? BMJ 1997;314:1037–40. 9112854 10.1136/bmj.314.7086.1037 PMC2126438

[2] Marmot MG. Understanding social inequalities in health. Perspect Biol Med 2003;46:S9–23. 14563071

[3] DeRouen MC , Hu L, McKinley M et al Incidence of lung cancer histologic cell-types according to neighborhood factors: a population based study in California. PLoS One 2018;13:e0197146. 29791458 10.1371/journal.pone.0197146 PMC5965814

[4] DeRouen MC , Schupp CW, Koo J et al Impact of individual and neighborhood factors on disparities in prostate cancer survival. Cancer Epidemiol 2018;53:1–11. 29328959 10.1016/j.canep.2018.01.003 PMC7499899

[5] Moss JL , Johnson NJ, Yu M, Altekruse SF, Cronin KA. Comparisons of individual- and area-level socioeconomic status as proxies for individual-level measures: evidence from the Mortality Disparities in American Communities study. Popul Health Metr 2021;19:1. 33413469 10.1186/s12963-020-00244-x PMC7792135

[6] Ingleby FC , Belot A, Atherton I, Baker M, Elliss-Brookes L, Woods LM. Assessment of the concordance between individual-level and area-level measures of socio-economic deprivation in a cancer patient cohort in England and Wales. BMJ Open 2020;10:e041714. 10.1136/bmjopen-2020-041714 PMC7692821 33243814

[7] Afshar N , English DR, Milne RL. Factors explaining socio-economic inequalities in cancer survival: a systematic review. Cancer Control J Moffitt Cancer Cent 2021;28:10732748211011956. 10.1177/10732748211011956 PMC8204531 33929888

[8] Narla NP , Pardo-Crespo MR, Beebe TJ et al Concordance between individual vs. area-level socioeconomic measures in an urban setting. HPU 2015;26:1157–72. 10.1353/hpu.2015.0122 26548670

[9] Buajitti E , Chiodo S, Rosella LC. Agreement between area- and individual-level income measures in a population-based cohort: implications for population health research. SSM—Popul Health 2020;10:100553. 32072008 10.1016/j.ssmph.2020.100553 PMC7013127

[10] Davis LE , Mahar AL, Strumpf EC. Agreement between individual and neighborhood income measures in patients with colorectal cancer in Canada. J Natl Cancer Inst 2023;115:514–22. 36708004 10.1093/jnci/djad017 PMC10165486

[11] Xie S , Hubbard RA, Himes BE. Neighborhood-level measures of socioeconomic status are more correlated with individual-level measures in urban areas compared with less urban areas. Ann Epidemiol 2020;43:37–43.e4. 32151518 10.1016/j.annepidem.2020.01.012 PMC7160852

[12] Jerrim J. *Measuring Socio-Economic Background Using Administrative Data. What is the Best Proxy Available?* DoQSS Work Pap. 2020; https://ideas.repec.org//p/qss/dqsswp/2009.html (5 September 2024, date last accessed).

[13] Booth CM , Li G, Zhang-Salomons J, Mackillop WJ. The impact of socioeconomic status on stage of cancer at diagnosis and survival: a population-based study in Ontario. Cancer 2010;116:4160–67. 20681012 10.1002/cncr.25427

[14] Berglund A , Garmo H, Robinson D et al Differences according to socioeconomic status in the management and mortality in men with high risk prostate cancer. Eur J Cancer 2012;48:75–84. 21852113 10.1016/j.ejca.2011.07.009

[15] Jembere N , Campitelli MA, Sherman M et al Influence of socioeconomic status on survival of hepatocellular carcinoma in the Ontario population; a population-based study, 1990–2009. PLoS One 2012;7:e40917. 22808283 10.1371/journal.pone.0040917 PMC3396620

[16] Johnson AM , Hines RB, Johnson JA, Bayakly AR. Treatment and survival disparities in lung cancer: the effect of social environment and place of residence. Lung Cancer Amst Neth 2014;83:401–407. 10.1016/j.lungcan.2014.01.008 24491311

[17] Petersen JM , Ranker LR, Barnard-Mayers R, MacLehose RF, Fox MP. A systematic review of quantitative bias analysis applied to epidemiological research. Int J Epidemiol 2021; 150:1708–30. 33880532 10.1093/ije/dyab061

[18] Lash TL , Fox MP, MacLehose RF, Maldonado G, McCandless LC, Greenland S. Good practices for quantitative bias analysis. Int J Epidemiol 2014;43:1969–85. 25080530 10.1093/ije/dyu149

[19] Fox MP , MacLehose RF, Lash TL. Applying Quantitative Bias Analysis to Epidemiologic Data. Springer Nature, 2022, p.475

[20] Radin RG , Rothman KJ, Hatch EE et al Maternal recall error in retrospectively-reported time-to-pregnancy: an assessment and bias analysis. Paediatr Perinat Epidemiol 2015;29:576–88. 26443987 10.1111/ppe.12245 PMC4651209

[21] Tjepkema M , Christidis T, Bushnik T, Pinault L. Cohort profile: The Canadian Census Health and Environment Cohorts (CanCHECs). Health Rep 2019;30:18–26. 31851369 10.25318/82-003-x201901200003-eng

[22] Government of Canada SC. *Canadian Cancer Registry (CCR)*. 2022. https://www23.statcan.gc.ca/imdb/p2SV.pl?Function=getSurvey&SDDS=3207 (5 September 2024, date last accessed).

[23] CIHI. *Trends in Income-Related Health Inequalities in Canada.* Ottawa, Ontario: Canadian Institutes of Health Information, 2015. https://secure.cihi.ca/free_products/trends_in_income_related_inequalities_in_canada_2015_en.pdf (5 September 2024, date last accessed).

[24] Wilkins R. *PCCF + Version 3G Users Guide: Automated Geographic Coding Based on the Statistics Canada Postal Code Conversion Files.* Cat. No. 82F0086-XDB. 2001. https://publications.gc.ca/site/eng/9.853482/publication.html.

[25] Statistics Canada. *Dissemination area: Detailed Definition*. https://www150.statcan.gc.ca/n1/pub/92-195-x/2011001/geo/da-ad/def-eng.htm (5 September 2024, date last accessed).

[26] Canadian Institutes of Health Information. *Measuring Health Inequalities: A Toolkit: Area-Level Equity Stratifiers Using PCCF and PCCF+*. Ottawa: Canadian Institutes of Health Information, 2018. https://www.cihi.ca/en/resources-for-measuring-health-inequalities (5 September 2024, date last accessed).

[27] Mendelson R. *Geographic Structures As Census Variables: Using Geography to Analyse Social and Economic Processes*. Ottawa: Statistics Canada, 2001. https://www150.statcan.gc.ca/n1/pub/92f0138m/92f0138m2001001-eng.pdf (5 September 2024, date last accessed).

[28] Statistics Canada. *Adjusted After-Tax Income of Economic Family*. 2016. https://www23.statcan.gc.ca/imdb/p3Var.pl?Function=DEC&Id=103386 (5 September 2024, date last accessed).

[29] Edge SB , Compton CC. The American Joint Committee on Cancer: the 7th edition of the AJCC cancer staging manual and the future of TNM. Ann Surg Oncol 2010;17:1471–74. 20180029 10.1245/s10434-010-0985-4

[30] Canadian Cancer Statistics Advisory Committee. *Canadian Cancer Statistics 2018*. Toronto, ON: Canadian Cancer Society, 2018. https://cdn.cancer.ca/-/media/files/research/cancer-statistics/2018-statistics/canadian-cancer-statistics-2018-en.pdf (5 September 2024, date last accessed).

[31] Fox MP , Lash TL, Greenland S. A method to automate probabilistic sensitivity analyses of misclassified binary variables. Int J Epidemiol 2005;34:1370–76. 16172102 10.1093/ije/dyi184

[32] Coughlin SS , Trock B, Criqui MH, Pickle LW, Browner D, Tefft MC. The logistic modeling of sensitivity, specificity, and predictive value of a diagnostic test. J Clin Epidemiol 1992;45:1–7. 1738006 10.1016/0895-4356(92)90180-u

[33] Banack HR , Stokes A, Fox MP et al Stratified probabilistic bias analysis for BMI-related exposure misclassification in postmenopausal women. Epidemiol Camb Mass 2018;29:604–13. 10.1097/EDE.0000000000000863 PMC6481627 29864084

[34] Fox J , Andersen R. Effect displays for multinomial and proportional-odds logit models. Sociol Methodol 2006;36:225–55.

[35] Chen W , Qian L, Shi J, Franklin M. Comparing performance between log-binomial and robust Poisson regression models for estimating risk ratios under model misspecification. BMC Med Res Methodol 2018;18:63. 29929477 10.1186/s12874-018-0519-5 PMC6013902

[36] Zou G. A modified poisson regression approach to prospective studies with binary data. Am J Epidemiol 2004;159:702–706. 15033648 10.1093/aje/kwh090

[37] Pichora E , Polsky JY, Catley C, Perumal N, Jin J, Allin S. Comparing individual and area-based income measures: impact on analysis of inequality in smoking, obesity, and diabetes rates in Canadians 2003–2013. Can J Public Health 2018;109:410–18. 29981091 10.17269/s41997-018-0062-5 PMC6964493

[38] Juhn YJ , Beebe TJ, Finnie DM et al Development and initial testing of a new socioeconomic status measure based on housing data. J Urban Health 2011;88:933–44. 21499815 10.1007/s11524-011-9572-7 PMC3191204

[39] Harris MN , Lundien MC, Finnie DM et al Application of a novel socioeconomic measure using individual housing data in asthma research: an exploratory study. NPJ Prim Care Respir Med 2014;24:14018. 24965967 10.1038/npjpcrm.2014.18 PMC4498187

[40] Bjur KA , Wi C-I, Ryu E et al Socioeconomic status, race/ethnicity, and health disparities in children and adolescents in a mixed rural-urban community—Olmsted County, Minnesota. Mayo Clin Proc 2019;94:44–53. 30611453 10.1016/j.mayocp.2018.06.030 PMC6360526

[41] Hammer R , Capili C, Wi CI, Ryu E, Rand-Weaver J, Juhn YJ. A new socioeconomic status measure for vaccine research in children using individual housing data: a population-based case-control study. BMC Public Health 2016;16:1000. 27655468 10.1186/s12889-016-3673-x PMC5031352

[42] Kim U , Koroukian SM, Stange KC, Spilsbury JC, Dong W, Rose J. Describing and assessing a new method of approximating categorical individual-level income using community-level income from the census (weighting by income probabilities). Health Serv Res 2022;57:1348–60. 35832029 10.1111/1475-6773.14026 PMC9643096

[43] Canadian Partnership Against Cancer. *Canadian Strategy for Cancer Control 2019–2029.* Toronto: Canadian Partnership Against Cancer; 2018.

[44] The NHS. *The NHS Long Term Plan*. London, UK, 2019. https://www.longtermplan.nhs.uk/wp-content/uploads/2019/08/nhs-long-term-plan-version-1.2.pdf (5 September 2024, date last accessed).

[45] European Commission. *Europe’s Beating Cancer Plan 2021–2030*. European Commission; 2021. https://health.ec.europa.eu/system/files/2022-02/eu_cancer-plan_en_0.pdf (5 September 2024, date last accessed).

