# Supplementary material for: Probabilistic bias analysis for exposure misclassification of household income by neighbourhood in a cohort of individuals with colorectal cancer
Source: Int J Epidemiol. 2024 Oct 13;53(6):dyae135. doi: 10.1093/ije/dyae135 (PMC11471264; doi:10.1093/ije/dyae135)
Supplement: dyae135_Supplementary_Data [file dyae135_supplementary_data.pdf]

Probabilistic bias analysis for exposure misclassification of household income by neighbourhood in a cohort of colorectal cancer patients

Laura E Davis, PhD, Hailey R Banack, PhD, Renzo Calderon-Anyosa MD, PhD, Erin C Strumpf, PhD, Alyson L Mahar PhD

Supplementary Tables

Table S1. Details on how to calculate crude positive predictive values (PPV) and negative predictive values (NPV)

Table S2. Comparison of study cohort to those excluded for missing postal code (could not be linked to the Postal Code Conversion File (PCCF+) to obtain neighbourhood income)

Table S3. Province stratified adjusted predictive values and 95% confidence intervals

Table S4. Unadjusted and adjusted bias-adjusted relative risks of death within five years (yes/no) with systematic and random error for all of Canada and by province, compared to the unadjusted and adjusted relative risk of death for true household income and measured neighbourhood income. The reference category is the highest income quintile for all comparisons (quintile 5)

Table S5. Cohort characteristics compared to all colorectal cancer patients diagnosed from 2006 to 2014 in the Canadian Cancer Registry (only compared on variables found in the CCR)

Table S1. Details on how to calculate crude positive predictive values (PPV) and negative predictive values (NPV)

		Household income quintiles					
		**Quintile 1 (lowest)**	**Quintile 2**	**Quintile 3**	**Quintile 4**	**Quintile 5 (highest)**	**Neighbourhood income quintile total**
**Neighbourhood income quintiles**	**Quintile 1 (lowest)**	**PPV1** 1440/4220 = 0.34	**NPV1.2** 1130/4220 = 0.27	**NPV1.3** 735/4220 = 0.17	**NPV1.4** 555/4220 = 0.13	**NPV1.5** 360/4220 = 0.09	4220
	**Quintile 2**	**NPV2.1** 955/4490 = 0.21	**PPV2** 1195/4490 = 0.27	**NPV2.3** 945/4490 = 0.21	**NPV2.4** 805/4490 = 0.18	**NPV2.5** 590/4490 = 0.13	4490
	**Quintile 3**	**NPV3.1** 795/4400 = 0.18	**NPV3.2** 1065/4400 = 0.24	**PPV3** 935/4400 = 0.21	**NPV3.4** 870/4400 = 0.20	**NPV3.5** 735/4400 = 0.17	4400
	**Quintile 4**	**NPV4.1** 600/4250 = 0.14	**NPV4.2** 850/4250 = 0.20	**NPV4.3** 920/4250 = 0.22	**PPV4** 940/4250 = 0.22	**NPV4.5** 940/4250 = 0.22	4250
	**Quintile 5**	**NPV5.1** 450/4245 = 0.11	**NPV5.2** 705/4245 = 0.17	**NPV5.3** 770/4245 = 0.18	**NPV5.4** 895/4245 = 0.21	**PPV5** 1425/4245 = 0.34	4245

Table S2. Comparison of study cohort to those excluded for missing postal code (could not be linked to the Postal Code Conversion File (PCCF+) to obtain neighbourhood income)

	**Excluded because missing postal code (N=5,170)**	**Study cohort (N=21,5600)**	**Standardized mean difference**
**Variables**			
**Age at diagnosis (mean (SD))**	69 (12)	66 (12)	0.071
**Sex**			
Male	2775 (53.7)	11930 (55.3)	0.033
Female	2395 (46.3)	9635 (44.7)	
**Tumour location**			
Rectal	1775 (34.3)	7230 (33.5)	0.016
Colon	3395 (65.7)	14330 (66.5)	
**Rural residence**			
Not rural	4275 (82.7)	16440 (76.3)	0.16
Rural	895 (17.3)	5120 (23.7)	
**Province at diagnosis**			
Atlantic provinces	430 (8.3)	2350 (10.9)	0.097
Quebec	810 (15.7)	3420 (15.9)	
Ontario	2145 (41.5)	8750 (40.6)	
Prairie provinces	965 (18.6)	3985 (18.5)	
British Columbia	820 (15.9)	3055 (14.2)	
**Diagnosis year**			
2006	635 (12.3)	2620 (12.2)	0.062
2007	680 (13.2)	2740 (12.7)	
2008	750 (14.5)	2955 (13.7)	
2009	710 (13.7)	2890 (13.4)	
2010	765 (14.8)	3000 (13.9)	
2011	405 (7.8)	1795 (8.3)	
2012	415 (8.0)	1865 (8.7)	
2013	390 (7.6)	1890 (8.8)	
2014	415 (8.0)	1805 (8.4)	
**Stage at diagnosis**			
0	15 (0.3)	80 (0.4)	0.06
I	415 (8.0)	2030 (9.4)	
II	550 (10.6)	2395 (11.1)	
III	635 (12.3)	2570 (11.9)	
IV	430 (8.3)	1790 (8.3)	
Unknown	100 (1.9)	455 (2.1)	
Missing	3030 (58.6)	12240 (56.8)	
**Household income quintile**			

Quintile 1	1470 (28.4)	4235 (19.6)	0.213
Quintile 2	1150 (22.3)	4930 (22.9)	
Quintile 3	880 (17.1)	4300 (19.9)	
Quintile 4	830 (16.0)	4065 (18.8)	
Quintile 5	840 (16.2)	4040 (18.7)	

Table S3. Province stratified adjusted predictive values and 95% confidence intervals
*Predictive values and confidence intervals obtained from multinomial models
*Adjusted predictive values for rural residence, age and sex (NB. The difference between crude and adjusted values were very small (<1%), therefore crude values are not presented)
*Abbreviations: Q=quintile; CI=confidence interval

	**Household income quintiles**																				
	**Count**						**Adjusted predictive values (95% CI)**														
**Canada**																					
**Neighbourhood income quintiles**							**Predictive value**	**Lower 95%**	**Upper 95%**	**Predictive value**	**Lower 95%**	**Upper 95%**	**Predictive value**	**Lower 95%**	**Upper 95%**	**Predictive value**	**Lower 95%**	**Upper 95%**	**Predictive value**	**Lower 95%**	**Upper 95%**
	**Survived 5 years (N=12,445)**																				
		**Q1**	**Q2**	**Q3**	**Q4**	**Q5**	**Q1**			**Q2**			**Q3**			**Q4**			**Q5**		
	**Q1**	710	590	405	325	205	28.54	23.26	34.47	24.51	19.56	30.24	19.43	14.96	24.85	14.42	10.57	19.37	13.11	9.44	17.92
	**Q2**	455	615	560	490	395	17.63	13.49	22.71	24.59	19.76	30.16	24.22	19.42	29.77	20.41	15.96	25.72	13.14	9.59	17.75
	**Q3**	415	575	560	540	470	15.77	11.86	20.65	23.32	18.58	28.85	25.15	20.23	30.80	18.23	13.98	23.43	17.53	13.37	22.64
	**Q4**	310	465	575	600	635	12.73	9.22	17.32	18.09	13.88	23.24	19.09	14.77	24.31	23.07	18.36	28.56	27.03	21.98	32.74
	**Q5**	220	370	455	560	950	11.84	8.31	16.59	13.70	9.91	18.63	18.45	14.08	23.82	17.70	13.42	22.98	38.31	32.38	44.61
	**Died within 5 years (N=9,150)**																				
		**Q1**	**Q2**	**Q3**	**Q4**	**Q5**	**Q1**			**Q2**			**Q3**			**Q4**			**Q5**		
	**Q1**	730	540	330	230	155	33.46	27.62	39.86	23.16	18.14	29.08	21.42	16.57	27.22	12.23	8.59	17.13	9.72	6.51	14.27
	**Q2**	500	575	385	315	195	24.39	19.02	30.69	25.61	20.15	31.95	20.34	15.43	26.31	17.86	13.27	23.60	11.81	8.14	16.83
	**Q3**	380	490	370	330	260	21.71	16.74	27.66	24.74	19.45	30.92	19.91	15.12	25.76	19.99	15.19	25.83	13.66	9.72	18.86
	**Q4**	290	385	345	345	305	25.42	19.34	32.63	21.74	16.10	28.68	14.16	9.59	20.40	21.27	15.63	28.25	17.42	12.33	24.04
	**Q5**	230	335	315	335	470	13.12	11.58	14.82	19.65	17.80	21.63	19.25	17.41	21.22	20.30	18.43	22.31	27.69	25.57	29.91
**Quebec**																					
**Neighbourhood income quintiles**							**Predictive value**	**Lower 95%**	**Upper 95%**	**Predictive value**	**Lower 95%**	**Upper 95%**	**Predictive value**	**Lower 95%**	**Upper 95%**	**Predictive value**	**Lower 95%**	**Upper 95%**	**Predictive value**	**Lower 95%**	**Upper 95%**
	**Survived 5 years (N=1,890)**																				
		**Q1**	**Q2**	**Q3**	**Q4**	**Q5**	**Q1**			**Q2**			**Q3**			**Q4**			**Q5**		
	**Q1**	135	95	50	45	40	35.04	30.23	40.17	26.49	22.16	31.32	14.70	11.38	18.79	12.54	9.48	16.42	11.23	8.35	14.96
	**Q2**	85	105	80	65	60	20.34	16.62	24.64	26.32	22.15	30.97	21.20	17.37	25.62	16.65	13.22	20.76	15.48	12.17	19.50

	**Q3**	70	95	75	90	85	16.94	13.60	20.91	22.97	19.13	27.32	18.22	14.77	22.26	21.49	17.78	25.74	20.38	16.76	24.56
	**Q4**	45	75	60	70	95	12.46	9.35	16.41	22.41	18.25	27.19	18.26	14.49	22.74	20.43	16.47	25.04	26.45	22.03	31.40
	**Q5**	35	50	70	95	120	9.11	6.55	6.55	14.32	11.06	11.06	19.48	15.70	15.70	25.39	21.16	21.16	31.71	27.10	27.10
	**Died within 5 years (N=1,525)**																				
		**Q1**	**Q2**	**Q3**	**Q4**	**Q5**	**Q1**			**Q2**			**Q3**			**Q4**			**Q5**		
	**Q1**	140	100	50	35	30	37.84	32.79	43.17	28.60	24.03	33.66	14.59	11.21	18.77	10.40	7.57	14.12	8.57	6.01	12.09
	**Q2**	90	100	70	50	30	25.80	21.40	30.75	30.30	25.61	35.44	20.58	16.58	25.25	15.15	11.69	19.40	8.17	5.68	11.61
	**Q3**	70	85	60	45	45	22.23	17.86	27.30	27.93	23.14	33.28	19.56	15.45	24.45	15.24	11.60	19.78	15.04	11.43	19.53
	**Q4**	45	60	60	55	50	16.57	12.50	21.63	22.82	18.12	28.32	22.73	18.07	28.18	20.35	15.92	25.64	17.53	13.42	22.56
	**Q5**	40	45	50	50	70	15.86	11.84	20.92	18.78	14.40	24.11	19.03	14.64	24.36	19.68	15.22	25.06	26.65	21.55	32.47
**Ontario**																					
							**Predictive value**	**Lower 95%**	**Upper 95%**	**Predictive value**	**Lower 95%**	**Upper 95%**	**Predictive value**	**Lower 95%**	**Upper 95%**	**Predictive value**	**Lower 95%**	**Upper 95%**	**Predictive value**	**Lower 95%**	**Upper 95%**
	**Survived 5 years (N=5,130)**																				
		**Q1**	**Q2**	**Q3**	**Q4**	**Q5**	**Q1**			**Q2**			**Q3**			**Q4**			**Q5**		
	**Q1**	265	235	175	140	65	29.97	27.00	33.13	27.11	24.24	30.18	19.97	17.43	22.77	15.69	13.42	18.27	7.26	5.73	9.15
	**Q2**	175	240	225	205	175	16.75	14.56	19.18	23.02	20.52	25.73	22.71	20.22	25.42	20.34	17.95	22.96	17.18	14.96	19.65
	**Q3**	150	235	230	230	185	14.27	12.25	16.55	22.71	20.23	25.39	22.61	20.14	25.29	22.61	20.14	25.29	17.80	15.57	20.28
**Neighbourhood income quintiles**	**Q4**	135	215	275	260	260	11.49	9.75	13.49	18.73	16.54	21.13	24.62	22.17	27.24	22.94	20.56	25.50	22.23	19.89	24.77
	**Q5**	80	165	180	230	400	7.27	5.86	8.99	15.12	13.07	17.42	17.34	15.15	19.77	22.34	19.90	24.98	37.93	35.01	40.95
	**Died within 5 years (N=3,630)**																				
		**Q1**	**Q2**	**Q3**	**Q4**	**Q5**	**Q1**			**Q2**			**Q3**			**Q4**			**Q5**		
	**Q1**	260	235	110	80	55	35.33	31.90	38.92	32.03	28.72	35.54	14.65	12.27	17.41	10.66	8.62	13.10	7.33	5.67	9.42
	**Q2**	195	230	150	130	90	24.06	21.19	27.19	29.31	26.21	32.61	18.98	16.37	21.89	16.80	14.33	19.60	10.85	8.86	13.22
	**Q3**	145	185	140	135	105	19.58	16.82	22.68	25.92	22.81	29.29	20.11	17.31	23.24	19.53	16.76	22.64	14.86	12.41	17.69
	**Q4**	110	160	150	140	130	15.92	13.35	18.87	23.69	20.63	27.06	22.35	19.36	25.65	20.52	17.64	23.73	17.52	14.85	20.55
	**Q5**	85	140	130	135	205	12.11	9.88	14.75	19.84	17.02	23.01	18.87	16.10	22.00	20.24	17.38	23.44	28.94	25.63	32.48
**British Colombia**																					
							**Predictive value**	**Lower 95%**	**Upper 95%**	**Predictive value**	**Lower 95%**	**Upper 95%**	**Predictive value**	**Lower 95%**	**Upper 95%**	**Predictive value**	**Lower 95%**	**Upper 95%**	**Predictive value**	**Lower 95%**	**Upper 95%**

	**Survived 5 years (N=1,755)**																				
		**Q1**	**Q2**	**Q3**	**Q4**	**Q5**	**Q1**			**Q2**			**Q3**			**Q4**			**Q5**		
	**Q1**	100	95	50	50	25	30.36	25.47	35.74	29.57	24.74	34.91	16.39	12.65	20.98	15.79	12.11	20.32	7.89	5.39	11.42
	**Q2**	55	95	75	65	55	15.71	12.24	19.95	27.67	23.17	32.67	22.64	18.50	27.40	18.49	14.70	23.00	15.49	12.02	19.74
	**Q3**	55	90	75	75	65	14.97	11.60	19.11	25.22	20.89	30.09	21.58	17.54	26.25	21.38	17.36	26.03	16.86	13.29	21.16
	**Q4**	50	50	70	100	95	13.75	10.56	17.72	14.20	10.95	18.22	19.43	15.64	23.88	27.19	22.82	32.05	25.43	21.16	30.23
**Neighbourhood income quintiles**	**Q5**	25	45	60	80	155	6.14	4.09	9.11	12.36	9.32	16.21	16.67	13.14	20.93	22.99	18.91	27.65	41.84	36.78	47.08
	**Died within 5 years (N=1,310)**																				
		**Q1**	**Q2**	**Q3**	**Q4**	**Q5**	**Q1**			**Q2**			**Q3**			**Q4**			**Q5**		
	**Q1**	95	70	55	50	20	33.11	27.84	38.85	24.40	19.74	29.77	18.86	14.70	23.86	17.08	13.11	21.94	6.55	4.22	10.01
	**Q2**	65	85	55	45	25	23.27	18.60	28.70	30.61	25.38	36.39	21.39	16.88	26.71	16.65	12.64	21.61	8.09	5.39	11.96
	**Q3**	45	65	55	40	30	17.99	13.55	23.48	29.06	23.54	35.28	24.02	18.94	29.97	17.85	13.43	23.33	11.08	7.68	15.73
	**Q4**	35	55	50	65	55	13.28	9.62	18.05	20.86	16.25	26.37	19.38	14.93	24.78	25.49	20.46	31.28	20.98	16.36	26.49
	**Q5**	25	45	50	55	75	9.40	6.30	13.80	18.21	13.82	23.62	20.66	15.97	26.29	21.91	17.11	27.60	29.83	24.32	35.99
**Atlantic provinces (Newfoundland, Prince Edward Island, Nova Scotia, New Brunswick)**																					
							**Predictive value**	**Lower 95%**	**Upper 95%**	**Predictive value**	**Lower 95%**	**Upper 95%**	**Predictive value**	**Lower 95%**	**Upper 95%**	**Predictive value**	**Lower 95%**	**Upper 95%**	**Predictive value**	**Lower 95%**	**Upper 95%**
	**Survived 5 years (N=1,315)**																				
		**Q1**	**Q2**	**Q3**	**Q4**	**Q5**	**Q1**			**Q2**			**Q3**			**Q4**			**Q5**		
	**Q1**	75	65	50	35	35	28.54	23.26	34.47	24.51	19.56	30.24	19.43	14.96	24.85	14.42	10.57	19.37	13.11	9.44	17.92
	**Q2**	50	65	65	55	35	17.63	13.49	22.71	24.59	19.76	30.16	24.22	19.42	29.77	20.41	15.96	25.72	13.14	9.59	17.75
**Neighbourhood income quintiles**	**Q3**	45	65	65	45	45	15.77	11.86	20.65	23.32	18.58	28.85	25.15	20.23	30.80	18.23	13.98	23.43	17.53	13.37	22.64
	**Q4**	35	50	50	60	75	12.73	9.22	17.32	18.09	13.88	23.24	19.09	14.77	24.31	23.07	18.36	28.56	27.03	21.98	32.74
	**Q5**	30	35	45	45	95	11.84	8.31	16.59	13.70	9.91	18.63	18.45	14.08	23.82	17.70	13.42	22.98	38.31	32.38	44.61
	**Died within 5 years (N=1,040)**																				
		**Q1**	**Q2**	**Q3**	**Q4**	**Q5**	**Q1**			**Q2**			**Q3**			**Q4**			**Q5**		
	**Q1**	80	55	50	30	25	33.46	27.62	39.86	23.16	18.14	29.08	21.42	16.57	27.22	12.23	8.59	17.13	9.72	6.51	14.27
	**Q2**	50	55	45	40	25	24.39	19.02	30.69	25.61	20.15	31.95	20.34	15.43	26.31	17.86	13.27	23.60	11.81	8.14	16.83
	**Q3**	50	55	45	45	30	21.71	16.74	27.66	24.74	19.45	30.92	19.91	15.12	25.76	19.99	15.19	25.83	13.66	9.72	18.86

	**Q4**	45	35	25	35	30	25.42	19.34	32.63	21.74	16.10	28.68	14.16	9.59	20.40	21.27	15.63	28.25	17.42	12.33	24.04
	**Q5**	30	45	40	40	35	15.64	11.08	21.60	23.47	17.90	30.13	21.20	15.91	27.66	21.03	15.76	27.50	18.67	13.67	24.96
**Prairie provinces (Manitoba, Saskatchewan, Alberta)**																					
**Neighbourhood income quintiles**							**Predictive value**	**Lower 95%**	**Upper 95%**	**Predictive value**	**Lower 95%**	**Upper 95%**	**Predictive value**	**Lower 95%**	**Upper 95%**	**Predictive value**	**Lower 95%**	**Upper 95%**	**Predictive value**	**Lower 95%**	**Upper 95%**
	**Survived 5 years (N=2,330)**																				
		**Q1**	**Q2**	**Q3**	**Q4**	**Q5**	**Q1**			**Q2**			**Q3**			**Q4**			**Q5**		
	**Q1**	130	100	80	55	40	32.71	28.27	37.48	23.98	20.04	28.41	20.66	16.96	24.94	13.53	10.51	17.25	9.12	6.69	12.31
	**Q2**	85	105	110	105	70	18.14	14.90	21.91	21.84	18.31	25.83	23.56	19.92	27.64	22.55	18.97	26.58	13.91	11.07	17.34
	**Q3**	90	85	115	95	90	18.79	15.50	22.60	17.47	14.29	21.19	24.81	21.09	28.95	20.36	16.93	24.28	18.56	15.26	22.38
	**Q4**	50	75	115	110	110	10.58	8.05	13.78	16.12	13.00	19.83	26.02	22.17	30.28	23.82	20.11	27.98	23.45	19.77	27.59
	**Q5**	50	75	100	110	180	10.14	7.79	13.10	14.07	11.30	17.38	20.01	16.73	23.76	21.59	18.20	25.41	34.19	30.14	38.48
	**Died within 5 years (N=1,655)**																				
		**Q1**	**Q2**	**Q3**	**Q4**	**Q5**	**Q1**			**Q2**			**Q3**			**Q4**			**Q5**		
	**Q1**	155	85	65	35	25	42.84	37.68	48.17	23.05	18.93	27.75	19.05	15.27	23.50	9.28	6.68	12.74	5.79	3.83	8.65
	**Q2**	100	105	70	50	30	26.96	22.48	31.96	30.36	25.68	35.48	20.77	16.78	25.43	13.51	10.27	17.56	8.41	5.92	11.81
	**Q3**	75	95	75	60	45	20.63	16.63	25.30	28.40	23.82	33.48	21.83	17.74	26.57	16.75	13.15	21.10	12.38	9.32	16.28
	**Q4**	55	70	65	50	45	18.79	14.56	23.90	25.87	20.98	31.43	23.86	19.15	29.31	16.93	12.94	21.85	14.55	10.88	19.21
	**Q5**	50	55	50	55	90	14.76	11.16	19.26	18.58	14.52	23.48	18.13	14.09	23.02	19.01	14.86	23.99	29.52	24.48	35.12

Table S4. Unadjusted and adjusted bias-adjusted relative risks of death within five years (yes/no) with systematic and random error for all of Canada and by province, compared to the unadjusted and adjusted relative risk of death for true household income and measured neighbourhood income. The reference category is the highest income quintile for all comparisons (quintile 5).

	**Unadjusted RR**			**Adjusted RR for age, sex, and rural residence**		
**Scenario**	**Simulation with random error (95% SI)**	**True household income (95% CI)**	**Measured neighbourhood income (95%CI)**	**Simulation with random error (95% SI)**	**True household income (95% CI)**	**Measured neighbourhood income (95% CI)**
**Canada**						
Income Quintile 1	1.42 (1.32-1.53)	1.46 (1.39-1.54)	1.18 (1.12-1.24)	1.36 (1.27-1.47)	1.26 (1.20-1.33)	1.14 (1.09-1.19)
Income Quintile 2	1.35 (1.25-1.45)	1.37 (1.30-1.44)	1.11 (1.05-1.16)	1.30 (1.21-1.40)	1.18 (1.12-1.24)	1.08 (1.03-1.13)
Income Quintile 3	1.17 (1.08-1.28)	1.19 (1.12-1.25)	1.05 (1.00-1.10)	1.15 (1.06-1.25)	1.09 (1.03-1.15)	1.04 (0.99-1.09)
Income Quintile 4	1.11 (1.02-1.20)	1.11 (1.05-1.18)	0.99 (0.94-1.04)	1.10 (0.96-1.23)	1.08 (1.02-1.14)	1.00 (0.95-1.05)
**Ontario**						
Income Quintile 1	1.39 (1.23-1.55)	1.42 (1.31-1.54)	1.15 (1.06-1.24)	1.35 (1.20-1.51)	1.23 (1.13-1.33)	1.14 (1.06-1.23)
Income Quintile 2	1.32 (1.18-1.48)	1.34 (1.24-1.45)	1.10 (1.02-1.19)	1.29 (1.15-1.44)	1.14 (1.06-1.23)	1.08 (1.00-1.16)
Income Quintile 3	1.11 (0.95-1.24)	1.10 (1.01-1.21)	1.03 (0.95-1.12)	1.09 (0.96-1.24)	1.01 (0.93-1.10)	1.02 (0.94-1.10)
Income Quintile 4	1.07 (0.95-1.19)	1.06 (0.97-1.16)	0.95 (0.88-1.04)	1.06 (0.85-1.28)	1.02 (0.93-1.11)	0.97 (0.90-1.05)
**Quebec**						
Income Quintile 1	1.35 (1.13-1.69)	1.42 (1.25-1.61)	1.19 (1.05-1.34)	1.31 (1.09-1.58)	1.26 (1.11-1.43)	1.11 (0.99-1.25)
Income Quintile 2	1.31 (1.11-1.62)	1.35 (1.19-1.53)	1.13 (1.00-1.28)	1.26 (1.05-1.51)	1.22 (1.08-1.38)	1.09 (0.99-1.25)
Income Quintile 3	1.24 (1.01-1.53)	1.28 (1.12-1.47)	1.03 (0.91-1.17)	1.21 (0.99-1.47)	1.23 (1.08-1.41)	1.02 (0.90-1.15)
Income Quintile 4	1.11 (0.88-1.41)	1.11 (0.96-1.28)	1.06 (0.93-1.21)	1.09 (0.75-1.44)	1.08 (0.94-1.25)	1.07 (0.94-1.22)
**British Colombia**						
Income Quintile 1	1.40 (1.13-1.71)	1.41 (1.23-1.63)	1.18 (1.04-1.34)	1.34 (1.10-1.64)	1.20 (1.04-1.38)	1.13 (1.00-1.28)
Income Quintile 2	1.34 (1.09-1.63)	1.36 (1.18-1.56)	1.09 (0.96-1.25)	1.29 (1.06-1.57)	1.16 (1.01-1.33)	1.04 (0.92-1.18)
Income Quintile 3	1.29 (1.04-1.60)	1.31 (1.13-1.51)	0.99 (0.86-1.14)	1.26 (1.01-1.54)	1.17 (1.02-1.35)	0.99 (0.87-1.13)
Income Quintile 4	1.19 (0.96-1.47)	1.21 (1.04-1.40)	1.02 (0.89-1.17)	1.18 (0.82-1.56)	1.13 (0.98-1.31)	1.02 (0.90-1.16)
**Prairie provinces**						
Income Quintile 1	1.52 (1.27-1.83)	1.59 (1.40-1.80)	1.28 (1.14-1.44)	1.45 (1.22-1.72)	1.37 (1.21-1.55)	1.22 (1.09-1.37)
Income Quintile 2	1.48 (1.24-1.78)	1.48 (1.30-1.68)	1.16 (1.03-1.30)	1.42 (1.19-1.69)	1.25 (1.10-1.41)	1.13 (1.01-1.27)
Income Quintile 3	1.20 (0.97-1.46)	1.18 (1.03-1.35)	1.15 (1.02-1.30)	1.17 (0.97-1.43)	1.09 (0.95-1.24)	1.14 (1.02-1.28)

Income Quintile 4	1.06 (0.84-1.31)	1.06 (0.91-1.22)	1.03 (0.91-1.18)	1.05 (0.75-1.38)	1.05 (0.91-1.22)	1.05 (0.93-1.19)
**Atlantic provinces**						
Income Quintile 1	1.49 (1.22-1.87)	1.54 (1.32-1.81)	1.10 (0.95-1.27)	1.44 (1.15-1.80)	1.38 (1.18-1.60)	1.08 (0.94-1.23)
Income Quintile 2	1.36 (1.13-1.74)	1.39 (1.18-1.63)	1.03 (0.89-1.19)	1.33 (1.05-1.67)	1.19 (1.02-1.40)	1.05 (0.91-1.20)
Income Quintile 3	1.25 (0.89-1.49)	1.24 (1.05-1.47)	1.05 (0.91-1.21)	1.21 (0.94-1.54)	1.15 (0.97-1.35)	1.06 (0.93-1.22)
Income Quintile 4	1.25 (0.99-1.61)	1.30 (1.10-1.54)	0.89 (0.76-1.04)	1.24 (0.84-1.68)	1.25 (1.06-1.48)	0.90 (0.78-1.05)

*****Abbreviations: RR = relative risk; CI = confidence interval; SI = simulation interval

Table S5. Study cohort characteristics compared to all colorectal cancer patients diagnosed from 2006 to 2014 in the Canadian Cancer Registry (only compared on variables found in the CCR)

	**CCR cohort diagnosed 2006-2014 (N=165,155)**	**Study cohort (21,595)**	**Standardized mean difference**
**Variabes**			
**Age at diagnosis (mean (SD))**	69.80 (12.62)	65.95 (12.46)	0.307
**diagnosis year**			
2006	19240 (11.7)	2620 (12.1)	0.099
2007	19930 (12.1)	2750 (12.7)	
2008	20505 (12.4)	2960 (13.7)	
2009	20670 (12.5)	2890 (13.4)	
2010	20835 (12.6)	3000 (13.9)	
2011	15755 (9.5)	1800 (8.3)	
2012	15860 (9.6)	1870 (8.7)	
2013	16060 (9.7)	1895 (8.8)	
2014	16300 (9.9)	1810 (8.4)	
**Sex**			
male	90655 (54.9)	11945 (55.3)	0.009
female	74505 (45.1)	9650 (44.7)	
**Tumour location**			
rectal	55090 (33.4)	7245 (33.6)	0.004
colon	110070 (66.6)	14350 (66.4)	
**Stage at diagnosis**			
0-1	16905 (10.2)	2115 (9.8)	0.064
2	18915 (11.5)	2400 (11.1)	
3	21375 (12.9)	2580 (11.9)	
4	15150 (9.2)	1790 (8.3)	
5	4030 (2.4)	460 (2.1)	
Missing/unknown	88785 (53.8)	12255 (56.7)	
**Province/territory at diagnosis**			
Atlantic provinces	17205 (10.5)	2350 (10.9)	0.043
Quebec	26370 (16.0)	3420 (15.8)	
Ontario	67885 (41.1)	8750 (40.5)	
Prairie provinces	28880 (17.5)	3985 (18.4)	
Territories	445 (0.3)	35 (0.2)	

*Abbreviations: CCR = Canadian Cancer Registry; PEI= Prince Edward Island
